# The Acute Effects of Heavy Deadlifts on Vertical Jump Performance in Men

**DOI:** 10.3390/sports4020022

**Published:** 2016-03-23

**Authors:** Jerry C. Arias, Jared W. Coburn, Lee E. Brown, Andrew J. Galpin

**Affiliations:** Center for Sport Performance, Department of Kinesiology, California State University, Fullerton, CA 92831, USA; titanedgeathletics@gmail.com (J.C.A.); leebrown@fullerton.edu (L.E.B.); agalpin@fullerton.edu (A.J.G.)

**Keywords:** postactivation potentiation, PAP, power

## Abstract

The purpose of this study was to investigate the effects of deadlifts as a postactivation potentiation stimulus on vertical jump performance. Fifteen men (age, 23.9 ± 4.2 years; height, 176.3 ± 8.6 cm; mass, 76.1 ± 16.3 kg) participated in the study. Participants visited the lab for three sessions, each separated by at least 48 h. One repetition maximum (1RM) in the deadlift was measured during the first visit. For Visit 2, participants performed one of two experimental sessions: a deadlift session or a control session. Participants performed a single maximal vertical jump (VJ; counter movement jump without an arm swing), then either performed five repetitions of the deadlift using 85% 1RM (deadlift session) or were told to stand still for ten seconds (control). Following either condition, participants performed single VJ at 15 s, 2, 4, 6, 8, 10, 12, 14, and 16 min post condition. Peak VJ height and peak ground reaction forces (pGRF) were measured using a force plate. For Visit 3, whatever condition was not administered at Visit 2 was performed. The results showed that VJ height was significantly lower 15 s following deadlifting (36.9 ± 5.1 cm) compared to the control condition (40.1 ± 4.6 cm). In addition, VJ height 15 s after the deadlift was lower than VJ height measured at minutes 2–16 following the deadlift. Performance of five repetitions of deadlifting did not affect pGRF. These results suggest that performing five repetitions of the deadlift exercise at 85% 1RM does not induce a postactivation potentiation (PAP) effect, and may in fact cause an acute reduction in VJ performance.

## 1. Introduction

Postactivation potentiation (PAP) is defined as an increase in muscle twitch and low-frequency tetanic force after a “conditioning” contractile activity [1]. It is believed that the potentiating effect from the conditioning activity may be due to phosphorylation of the myosin regulatory light chains, or increased excitability of alpha motor neurons in the spinal cord [2]. The “conditioning” activity that has been used in past research to evoke PAP has included both dynamic [3,4,5] and isometric [6,7] muscle actions. Past research has shown PAP to be effective in producing greater performance in high intensity activities such as jumping and sprinting [8,9,10,11]. However, there is also evidence to suggest that PAP has no significant effect on performance variables [4,12,13,14,15] or may actually impair performance [16,17]. The latter may result when fatigue from the conditioning activity is greater than the potentiating effect [2].

Differences between studies, such as exercise selection, intensity, type of muscle contraction, rest periods following the potentiating activity, subject characteristics (age, strength, sex), and the variables being measured may all contribute to such variability in results. For example, it has been demonstrated that trained individuals may be more responsive to a potentiating stimulus than untrained individuals [18], at least partly due to differences in absolute and/or relative strength. The time from the PAP stimulus until performance is assessed has also affected the interpretation of the effectiveness of PAP stimuli. For example, some studies have reported positive effects 4 to 12 min following the PAP stimulus [9], but no positive effect at 10 [4] or 15 s [9]. However, at least one other study did report increases in jump performance 20 s and four minutes following isometric squats in adult male participants [19]. Thus, further research into the timing of an optimal PAP effect is warranted.

When selecting an exercise to induce a PAP stimulus, it is important to select one that is both effective and practical to use. While previous studies have examined the use of exercises such as the back squat to elicit a PAP effect [3,4,9,20], there may be similar but more effective exercises. The deadlift exercise was chosen for the present study because it is a relatively simple exercise to perform, and requires similar muscle actions to that of a vertical jump (hip and knee extension). From a practical standpoint, it requires minimal equipment. If an athlete wanted to take advantage of a PAP response out on the field or court prior to competition, all that would be needed would be a heavy object to lift off the ground. Therefore, the purpose of this study was to investigate the effects of heavy deadlifts on vertical jump (VJ) and ground reaction forces (GRF) in resistance trained men.

## 2. Methods

### 2.1. Participants

Fifteen (age = 23.9 ± 4.2 years; height = 176.3 ± 8.6 cm; body mass = 80.1 ± 10.2 kg; deadlift 1RM = 152.4 ± 27.9 kg; relative deadlift 1RM = 1.9 ± 0.2 kg·kg^−1^) men volunteered to participate in this study. Prior to testing, participants signed a statement of informed consent approved by the University Institutional Review Board. All participants met the following inclusion criteria: male between 18 and 35 years, performed at least one lower body resistance exercise session per week for the past six months, and free of injuries that would limit their ability to perform maximal deadlifts or jumping exercises. Participants were instructed to refrain from any type of resistance training that involved the lower body 48 h prior to testing, maintain a normal diet, and drink half a liter of water on test day to ensure proper hydration.

### 2.2. Experimental Design

Three laboratory sessions, separated by at least 48 h, were required for each participant. The first session was an orientation/familiarization visit. During the orientation session, participants were asked to complete the following: informed consent, measurements of height and weight, maximum vertical jump height, and 1RM testing in the deadlift. The other two sessions were either the deadlift or control session. During these two sessions, participants were required to do a countermovement jump for determination of VJ height, followed by either the treatment (85% 1RM deadlift for five repetitions) or control (no deadlift). The order of these sessions were randomized for the first participant and counterbalanced for each remaining participant. These sessions began with a dynamic warm-up, which included twenty jumping jacks, ten walking T-Dives, ten walking lunges, and ten repetitions of the deadlift with a 45 lb. Olympic bar. The warm-up was followed by five minutes of sitting rest. Following the brief rest period, subjects either completed the control or treatment protocol. This was followed by single countermovement jumps at 15 s, 2, 4, 6, 8, 10, 12, 14, and 16 min post deadlift or control period. Participants were resting in a sitting position between the jumps. The use of an 85% 1RM load in the deadlift was chosen because it had previously been shown that the use of maximal or near maximal intensity (>80%) has greater effects at inducing PAP when followed by an explosive activity such as jumping [10,21].

### 2.3. 1RM Testing

The 1RM testing procedure began with a warm up of five to ten repetitions at 40% to 60% of estimated 1RM. After a one minute rest, the participant performed three to five repetitions at 60%–80% of perceived maximum (moderate-to-hard exertion). The participant then attempted a 1RM lift. If the lift was successful, a rest period of 3–5 min was provided before attempting a heavier weight. The goal was to find the 1RM within three to five maximal efforts. The process of increasing the weight was continued by increments of approximately 7.5 to 15 kg, until the participant was able to only complete one repetition successfully. If the participant was not able to execute the lift successfully, the weight was reduced by 2.2 to 4.5 kg. A Certified Strength and Conditioning Specialist certified through the National Strength and Conditioning Association oversaw each attempt. A deadlift 1RM attempt was deemed successful if at the end of the ascent phase the participant stood erect with knees and hips extended, the torso upright, and the should girdle retracted.

### 2.4. Vertical Jump and Ground Reaction Force Testing

Subjects performed countermovement jumps on a force platform. The countermovement jumps were performed in accordance with Young *et al.* [22] and similar to the procedures to that of Till & Cook [12]. Participants began in a standing position with hands positioned on their hips, then flexing the hips and knees, and immediately jumping vertically as high as possible. The force plate was used to estimate VJ height (cm) using the time in the air equation: distance = (1/2 gt^2^)/2, where g is gravity at 9.81 m·s^2^ and t is the flight time. Measurements of VJ height (primary outcome variable) and peak ground reaction forces (pGRF; secondary outcome variable) were collected immediately following each jump. VJ height and pGRF were measured on a force plate (AMTI force platform, Advanced Mechanical Technology, Inc., Watertown, MA, USA). Data were collected at a sampling frequency of 1000 Hz using a computer and custom LabVIEW version 7.1 software (National Instruments Corporation, Austin, TX, USA). Verbal encouragement was given during both conditions to encourage maximal performance.

### 2.5. Statistical Analyses

All statistical analyses were conducted using IBM SPSS Statistics 20.0 (IBM Corporation, Somers, NY, USA). A 2 × 10 (condition × time) repeated measured analysis of variance (ANOVA) was performed for each dependent variable (VJ height and pGRF). Significant interactions were followed up with one-way repeated measures ANOVAs, Tukey post hoc tests, and paired *t*-tests as appropriate. Alpha was set at 0.05. Effect sizes were calculated by SPSS as partial eta squared (η_p_^2^ = SS_effect_/(SS_effect_ + SS_error_).

## 3. Results

**Figure 1:**
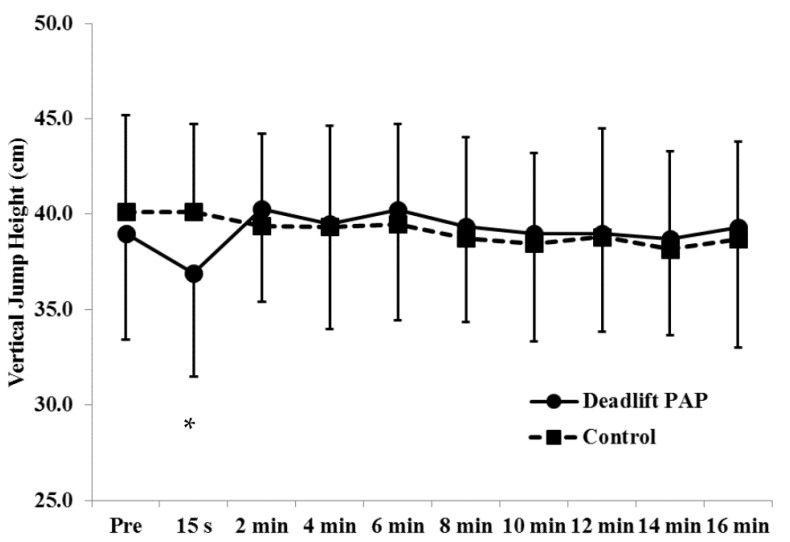
Vertical jump (VJ) height (mean ± SD). There was a significant 2-way (treatment × time) interaction (*p* < 0.05). The deadlift condition led to a significant decrease in vertical jump height 15 s after deadlifting compared to subsequent time periods. * = less than 2, 4, 6, 8, 10, 12, 14, and 16 min. In addition, VJ height at 15 s was significantly lower for the deadlift condition compared to the control condition.

For VJ height, there was a significant 2-way (treatment × time) interaction (*p* < 0.05). For the deadlift condition, follow-up tests indicated VJ height at 15 s post deadlift was significantly (*p* < 0.05) lower than VJ height at 2, 4, 6, 8, 10, 12, 14, and 16 min (Figure 1). For the control condition, there was no significant main effect for time (*p* > 0.05). In addition, VJ height at 15 s was significantly lower for the deadlift condition compared to the control condition.

**Figure 2:**
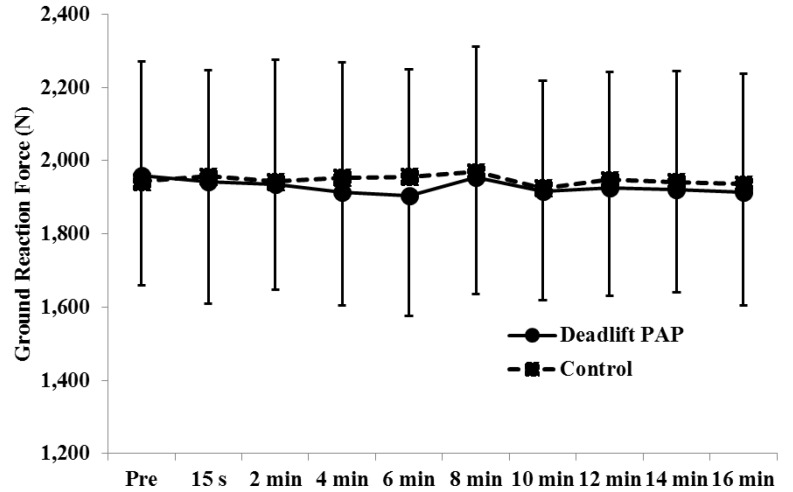
Ground reaction force (mean ± SD). There was no significant interaction or main effects (*p* > 0.05).

For pGRF, there was no significant (*p* > 0.05) two-way (treatment × time) interaction or main effects for time (Figure 2). Effect sizes for VJ height and pGRF were interpreted as trivial to small effects (Table 1).

## 4. Discussion

The purpose of this study was to determine the potentiating effects of heavy deadlifts on VJ height and pGRF. It was hypothesized that VJ height and pGRF would both improve following the deadlifts compared to the control condition. However, heavy deadlifts decreased VJ height 15 s post lifting compared to subsequent time periods. Furthermore, there were no significant effects of heavy deadlifts on pGRF.

Although the movement patterns during the deadlift exercise are similar to the VJ (extension of the hip, knee, and ankle joints), they are different with regard to joint ranges of motion. To our knowledge, only one other study has used the deadlift exercise as a conditioning stimulus to achieve a PAP effect [12]. They had subjects perform the deadlift for five repetitions at their 5RM. They did not find any significant improvements in either sprint times or vertical jump performance measures. Although they used different methodologies than the present study, both studies failed to find improved performance. Previous research has shown that the biomechanics of vertical jumping are altered in different ways by Olympic weightlifting, plyometrics, and a combination of the two [23]. This may also be true when considering the acute effects of heavy deadlifts, as used in the present study, compared to various forms of squatting as used in most other studies examining PAP. Thus, exercise selection may influence the effectiveness of the conditioning stimulus. Future studies should directly compare various PAP exercises, including the deadlift and back squat variations, to determine their relative effects on the PAP response.

Previous studies have shown improvements in vertical jump performance following back squats [5,22,24]. For example, Young *et al.* [22] examined the effects of using 5RM half-squats on loaded counter-movement jumps (LCMJ), and found that the LCMJ was 2.8% higher following half-squats. Lowery *et al.* [20] also reported significant increases in vertical jump four minutes following moderate (70% 1RM) and high intensity (93% 1RM), but not low intensity (56% 1RM), back squat loads. Vertical jump height was reported to have returned to baseline by minutes 8 and 12. Furthermore, they reported decreases in vertical jump height when measured immediately following the back squats using the moderate and high intensity loads. These studies are in contrast to the findings of Mangus *et al.* [3], who found no potentiating effects of half and quarter-squats on vertical jump performance. Although the results of our study may have been attributed to the use of the selected exercise (deadlift), the literature suggests that even when the same exercise is used in different studies, the results are inconsistent.

The timing of the jumps after the attempted PAP stimulus may also have contributed to the findings of the present study. There is evidence to suggest that fatigue and potentiation can coexist in skeletal muscle [25]. It has been suggested that fatigue may be dominant in the early stages of recovery, and thus, may hinder performance of subsequent voluntary activity [26]. In the present study, jumps were performed at 15 s, 2, 4, 6, 8, 10, 12, 14, and 16 min after the potentiating stimulus. Our finding of a reduction in VJ at 15 s suggests that fatigue was dominant immediately following the deadlift. Jensen and Ebben [4] also found no significant improvements in VJ height with similar rest periods (10 s, 1, 2, 3, and 4 min) following a 5RM back squat. Crewther *et al.* [9] examined the acute potentiating effects of back squats on the CMJ, sprint performance, and horizontal sled pushes. Their results indicated a significant reduction in CMJ height at 15 s, followed by improvements at 4, 8, and 12 min, and then a further decrease in CMJ performance at 16 min. Thus, performance may be significantly hindered when jumps are performed immediately after the potentiating stimulus. Arabatzi *et al.* [19], however, found that squat jump performance was enhanced both 20 s and four minutes following the performance of 3 × 3 maximal isometric squats. This was true only for the adult male participants, however. Preadolescent and adolescent males and females, and adult females, did not demonstrate a PAP effect during the squat jumps. While the higher absolute loads used by stronger individuals might allow the PAP effect to last for a longer period of time, it would also likely cause greater fatigue to be evident during the early phase of recovery following the PAP stimulus [20]. In our study, the 85% 1RM load used for the PAP stimulus may have induced significant fatigue, but was not enough to induce a PAP effect sufficient to lead to increases in VJ performance as the fatigue subsided. Once again, these studies illustrate the complex interplay of age, sex, type of conditioning stimulus, absolute and relative strength, and timing of assessment in determining the effectiveness of a conditioning stimulus at inducing a PAP effect [20].

Interestingly, both the Crewther *et al.* [9] and Jensen and Ebben [4] studies measured jumps at the four minute interval, yet only Crewther *et al.* showed significant improvements. Jensen and Ebben [4] measured consecutive jumps in one minute intervals, while our study used two minute intervals, and both failed to show significant improvements. However, Crewther *et al.* [9] allowed 4 min to pass between jumps and found significant improvements in jump height at 4, 8, and 12 min. This indicates there may be an ideal time, somewhere between 4 and 12 min, in which fatigue dissipates enough to allow the potentiation response to be evident. With regard to the present study, perhaps we would have observed a potentiating effect if we had begun vertical jump testing at four minutes after the conditioning activity, rather than including prior test jumps at 15 s and 2 min following the deadlifts. The jumps prior to the 4 min time period may have further fatigued, or delayed the recovery of, our subjects, masking the PAP response. This evidence supports the hypothesis that fatigue and potentiation can coexist, and in order to induce PAP, one must allow enough time to elapse to see any performance enhancement.

The strength level of the subjects may also have affected the PAP response [26]. Subjects who are stronger have been shown to have a greater potentiation response when compared to weaker individuals [22,27]. Chiu *et al.* [27] investigated the response to postactivation potentiation between athletes involved in sports requiring explosive strength, and recreationally trained subjects. Their results showed that athletes exhibited greater potentiation compared to recreationally trained individuals. Likewise, Young *et al.* [22] showed that subjects who had the greatest increase in mean jump height following the squat set, also achieved the greatest 5RM. They concluded that the stronger the individual, the greater the gain in power through PAP. It is possible that because we used recreationally trained men, and not athletes, the participants may not have been strong enough to benefit from the PAP stimulus (mean 5RM load, 129.7 ± 23.5 kg.; mean deadlift 1RM, 152.4 ± 27.9 kg; relative deadlift 1RM = 1.9 ± 0.2 kg·kg^−1^). The importance of absolute *vs.* relative strength must also be considered. Although few studies have reported relative strength values for their subjects, it seems reasonable that this may be at least as important as absolute strength in determining the effectiveness of a PAP stimulus on vertical jump height and power [28].

## 5. Conclusions

In conclusion, the lack of a positive PAP effect of deadlifts on VJ height or pGRF may be attributed to three main findings. First, the deadlift exercise may not have had enough specificity to the CMJ. Perhaps the back squat or jump squat would have yielded improvements in performance. Secondly, our results may have been affected by the recovery time chosen. The present study allowed 2 min in between jumps, and just 15 s following the PAP stimulus. It is possible that this was not enough time to recover and allow fatigue to dissipate. We can infer that if more time was given prior to the first jumps performed after the deadlifts, potentiation may have been evident and subsequent performance might have increased. Lastly, using only recreationally trained subjects, and not a stronger population of athletes, may have contributed to the lack of PAP response. In the present study, the subjects were required to have performed only one lower body resistance exercise session per week for the past six months. Therefore, our participants may not have been strong enough (absolute or relative strength) or trained enough to be able to induce the PAP response necessary to achieve an increase in vertical jump performance. The evidence from previous studies supports the contention that stronger individuals will benefit more from PAP methods than recreationally trained individuals.

Based on these results, the use of heavy deadlifts, as performed in this study, may not be sufficient to induce PAP in recreationally trained individuals, and may in fact reduce performance for several minutes due to fatigue. However, the present study also used relatively short rest periods following the PAP stimulus. If strength and conditioning practitioners wish to use deadlifts as a PAP stimulus to increase their athlete’s performance, they may want to allow somewhere between 4 and 12 min between the conditioning stimulus and the performance activity before assessing their effects.

## Figures and Tables

**Table 1:** Effect sizes (calculated as partial eta squared) for the dependent variables.

Variable	Effect Size (Partial Eta Squared)
VJ Height (deadlift condition)	0.226
VJ Height (control condition)	0.153
pGRF (deadlift condition)	0.067
pGRF (control condition)	0.025

Effect sizes for dependent variables. The numbers represent the proportion of the total variance that was due to the treatment (either deadlift or control condition). pGRF = peak ground reaction forces.
